# The Significance of Entropy in Grain Boundary Segregation

**DOI:** 10.3390/ma12030492

**Published:** 2019-02-05

**Authors:** Pavel Lejček, Siegfried Hofmann, Václav Paidar

**Affiliations:** 1 Institute of Physics, Academy of Sciences of the Czech Republic, Na Slovance 1999/2, 182 21 Prague 8, Czech Republic; paidar@fzu.cz; 2 Max-Planck-Institute for Intelligent Systems, Heisenbergstr. 3, 70569 Stuttgart, Germany; s.hofmann@is.mpg.de

**Keywords:** grain boundaries, solute segregation, thermodynamics, entropy, materials properties

## Abstract

The role of entropy in materials science is demonstrated in this report in order to establish its importance for the example of solute segregation at the grain boundaries of bcc iron. We show that substantial differences in grain boundary chemistry arise if their composition is calculated with or without consideration of the entropic term. Another example which clearly documents the necessity of implementing the entropic term in materials science is the enthalpy-entropy compensation effect. Entropy also plays a decisive role in the anisotropy of grain boundary segregation and in interface characterization. The consequences of the ambiguous determination of grain boundary segregation on the prediction of materials behavior are also briefly discussed. All the mentioned examples prove the importance of entropy in the quantification of grain boundary segregation and consequently of other materials properties.

## 1. Introduction

Grain boundary segregation is a phenomenon that influences the behavior of the whole material under external conditions [1]. By affecting the chemical composition of interfaces, grain boundary segregation has important consequences; it can evoke, for example, materials embrittlement due to a reduction of cohesion at the interfaces [2], or it can stabilize the nanocrystalline structure through reduced mobility of the grain boundaries [3]. Due to these consequences, grain boundary segregation has been continually studied through the decades [1]. These studies provide us, on the one hand, with experimental data on the chemical composition of grain boundaries in various binary as well as multicomponent polycrystalline systems, on the temperature dependence of the grain boundary composition in polycrystals, and on defined grain boundaries in bicrystals [1,4]. On the other hand, substantial progress has been made in the last decades in the theoretical calculations of grain boundary segregation in binary systems that provide us with segregation energies for individual sites of chosen grain boundaries [5].

Experimental data on grain boundary segregation have been most frequently obtained from measurements by Auger electron spectroscopy (AES) but recently also from analytical transmission electron microscopy or 3D atom probe tomography [5]. The temperature dependence of the grain boundary composition obtained in this way can then be correlated with a suitable model to obtain the values of the enthalpy and entropy of segregation of the solute studied. Besides the traditional models based on segregation isotherms of the Langmuir-McLean type [1], new approaches to describe the grain boundary segregation appear in literature such as the most recent report by Kaptay [6]. Once the values of segregation enthalpy and entropy—and in some cases those of the interaction parameters—are known, the grain boundary composition can be predicted by systems which have not yet been studied experimentally.

Thermodynamic quantities have been calculated by theoretical models such as density functional theory (DFT), molecular statics and dynamics, and the Monte Carlo methods (for a review, see Reference [5]). However, calculations by DFT are most frequently performed at 0 K. Therefore, they only provide us with the values of segregation energy ([7]) while segregation entropy cannot be principally obtained in this way. Nevertheless, several papers appeared in the literature that predict grain boundary composition in binary systems with varied bulk concentration in a broad temperature range without consideration of the entropy term (e.g., [8,9,10,11]), despite frequent indications ([1]) that entropy is a significant parameter in various phenomena of materials science. The question is then, is the entropic contribution important for the determination of the grain boundary concentration of a solute (and consequently, for the prediction of materials properties), or can it simply be ignored? 

In the present paper, it is discussed whether the differences caused by neglecting the entropic contribution are important for the determination of the grain boundary solute concentration or not. Other aspects of the entropic term, such as the enthalpy-entropy compensation effect and the reversed anisotropy of grain boundary segregation, are discussed. The effects of an incorrect determination of the grain boundary segregation on related materials properties are also briefly listed. Based on all findings, it is shown that the entropy of grain boundary segregation is an important parameter that cannot be neglected.

## 2. Thermodynamics of Grain Boundary Segregation

As mentioned above, the most popular expression for the description of solute concentration at the grain boundary is the Langmuir–McLean segregation isotherm,

(1) $$\frac{X_{I}^{GB}}{X^{0}-X_{I}^{GB}}=\;\frac{X_{I}^{v}}{1-X_{I}^{v}}\exp\left(-\frac{\Delta G_{I}}{RT}\right),$$

where $X_{I}^{GB}$ and $X_{I}^{v}$ are the concentrations of solute *I* at the grain boundary and in the volume of the host material, *M*, respectively. $X^{0}$ is the limit of saturation of the solute at the grain boundary, and

(2) $$\Delta G_{I}=\Delta G_{I}^{0}+\Delta G_{I}^{E}$$

is the Gibbs energy of segregation. It consists of two parts, the standard (ideal) Gibbs energy, $\Delta G_{I}^{0}$, and the excess Gibbs energy, $\Delta G_{I}^{E}$, representing the real contribution to $\Delta G_{I}$ [1]. In binary systems, the excess term has been most frequently correlated according to the simplified Fowler model [1],

(3) $$\Delta G_{I}^{E}=-2\alpha_{I(M)}(X_{I}^{GB}-X_{I}^{v}),$$

where $\alpha_{I\left(M\right)}$ is the coefficient of binary *I*-*I* interaction in the host material *M*. The standard term is constructed by the enthalpy (*H*) and entropy (*S*) terms,

(4) $$\Delta G_{I}^{0}=\Delta H_{I}^{0}-T\Delta S_{I}^{0}.$$

From the experimentally measured temperature dependence of the chemical composition of the grain boundaries, the values of all three parameters required for a complete description of grain boundary segregation, i.e., $\Delta H_{I}^{0}$, $\Delta S_{I}^{0}$, and $\alpha_{I\left(M\right)}$, can be obtained. 

Theoretical calculations result in the values of the Helmholtz energy of solute segregation, Δ*F_I_*. There is a simple thermodynamic relationship between $\Delta F_{I}$ and $\Delta G_{I}$,

(5) $$\Delta G_{I}=\Delta F_{I}+P\Delta V_{I}.$$

In Equation (5), *P* is pressure and $\Delta V_{I}$ is the segregation volume. It was recently shown that the term $P\Delta V_{I}$ is rather small and can be neglected, so that $\Delta F_{I}$ and $\Delta G_{I}$ are nearly equal under normal pressure [12],

(6) $$\Delta G_{I}≅\Delta F_{I}.$$

At *T* = 0 K, the entropic term is zero and $X_{I}^{GB}$ reaches the maximum value, i.e., $X^{0}$. Equation (6) can be thus rewritten as

(7) $$\Delta H_{I}^{0}-2\alpha_{I\left(M\right)}\left(X^{0}-X_{I}^{v}\right)≅\Delta E_{I},$$

where $\Delta E_{I}$ is the internal energy of the grain boundary segregation of *I* in *M*. Equation (7) represents the basis for comparison of the theoretical data with the experimental results [5]. 

## 3. Effect of Entropy in Grain Boundary Segregation

### 3.1. Temperature Dependence

A considerable number of published values of segregation energy and enthalpy exist. These values have been obtained either theoretically or experimentally, and much of the available data for selected host materials has been summarized in References [1,5], where the advantages and disadvantages of theoretical and experimental approaches are also discussed. 

**Figure 1:**
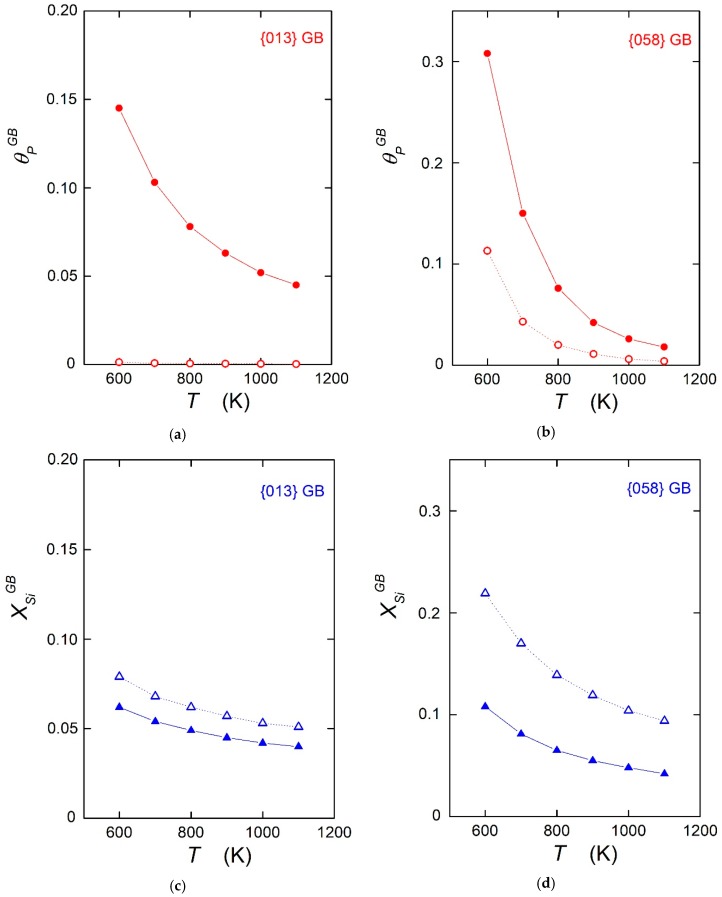
Calculated temperature dependences of grain boundary segregation with (solid symbols) and without (empty symbols) consideration of the segregation entropy according to Equation (1). (**a**,**b**): phosphorus (for $X_{P}^{v}$ = 0.0001), (**c**,**d**): silicon (for $X_{Si}^{v}$ = 0.03); (**a**,**c**): {013} special grain boundary, (**b**,**d**): {058} general grain boundary. $\theta_{P}^{GB}=X_{P}^{GB}/X^{0}$, $X^{0}$ = 2/3. Data are from Reference [13].

A comparison of experimental results and theoretical calculations on the values of the enthalpy and/or energy of grain boundary segregation found in the literature shows excellent agreement between cases where all physical prerequisites are fulfilled [5,13,14]. Let us note that this comparison is limited exclusively to energetic variables according to Equation (7), as the calculations were conducted at 0 K. However, some authors use the data on segregation energy obtained at 0 K for extrapolation to higher temperatures to determine the temperature dependence of the grain boundary concentration according to Equation (1) [8,9,10,11]. In fact, this means that the entropy contribution is neglected, and the calculated $\Delta E_{I}$ is used in these model extrapolations instead of $\Delta F_{I}$ Unfortunately, this negligence may result in significant ambiguity. This ambiguity is documented here, for example, in the segregation of phosphorus and silicon in α-iron. In Table 1, the data given were determined from measurements of the temperature dependence of grain boundary composition by AES for two grain boundaries, {013} and {058} [14]. These grain boundaries are representatives for special and general interfaces, respectively, which differ in their behavior and in the values of segregation enthalpy, but also in the values of segregation entropy (i.e., maximum and minimum values in the latter case, respectively). The temperature dependence of phosphorus ($X_{P}^{v}$ = 0.0001) and silicon ($X_{Si}^{v}$ = 0.03) segregation was calculated according to Equation (1), with and without consideration of segregation entropy. The results of these calculations are shown in Figure 1 for the temperature range of 600–1100 K, which are the most important temperatures for the application of iron and steels.

It is obvious from Figure 1 that substantial differences exist between the calculated values of $X_{I}^{GB}$ and enrichment, $\theta_{I}^{\;GB}=X_{I}^{GB}/X^{0}$, performed with and without consideration of segregation entropy. Maximum differences occur in the case of phosphorus segregation at low temperatures, as shown in Figure 1. With decreasing temperatures, the differences between the grain boundary concentrations determined with and without segregation entropy for the same grain boundary decrease according to Equation (1). However, these differences are still large even close to the α → γ transformation temperature (1184 K). It is also clear from Figure 1 that the grain boundary concentration of the solute is affected by the sign of segregation entropy. If the value of $\Delta S_{I}^{0}$ is negative, the concentrations calculated without consideration of entropy are higher than those calculated with $\Delta S_{I}^{0}$, as shown in Figure 1c,d, and vice versa in Figure 1a,b. 

### 3.2. Enthalpy-Entropy Compensation Effect

**Figure 2:**
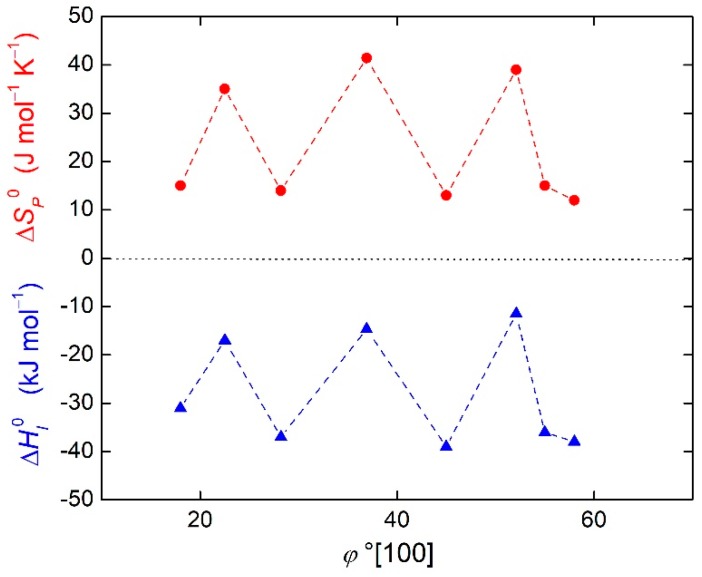
Orientation dependence of enthalpy (triangles) and entropy (circles) of phosphorus segregation at [100] symmetrical tilt grain boundaries of α-iron. Data are from Reference [14].

The importance of entropy in grain boundary segregation was already demonstrated in the case of the enthalpy-entropy compensation effect in Reference [15]. In fact, the orientation dependences of segregation entropy and enthalpy are very similar, as shown in Figure 2 for phosphorus at [100] symmetrical tilt grain boundaries of α-iron [16]. This fact can be expressed by the following equation:

(8) $$d\Delta H_{I}^{0}\left(\Phi \right)≅T_{CE}d\Delta S_{I}^{0}\left(\Phi \right),$$

where the differential is related to the changes of the grain boundary structure, Φ. The integration of Equation (8) results in

(9) $$\Delta S_{I}^{0}\left(\Phi \right)≅\frac{\Delta H_{I}^{0}\left(\Phi \right)}{T_{CE}}+\Delta S^{\prime }.$$

**Figure 3:**
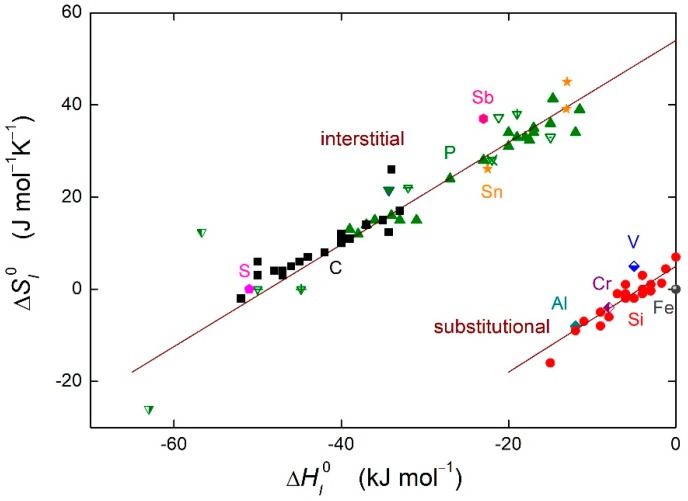
Enthalpy-entropy compensation effect for solute segregation in α-iron. Solid symbols: defined grain boundaries in bicrystals [14], with permission from © IOP Publishing. Other symbols: grain boundaries in polycrystals (data summarized in Reference [1]). Upper branch represents interstitial segregants in α-iron, bottom branch represents substitutional segregation.

In Equations (8) and (9), *T_CE_* is the compensation temperature and $\Delta S^{\prime }$ is the integration constant. The integral form of the enthalpy-entropy compensation effect (Equation (9)) is depicted in Figure 3 for the grain boundary segregation in α-iron. It can be seen clearly in Figure 3 that the linear dependence between the entropy and enthalpy of the grain boundary segregation is fulfilled in α-iron. The dependence is split into two branches, one for the interstitial segregants, the other one for the substitutional segregants [15]. 

The existence of the enthalpy-entropy compensation effect has another very important consequence. The combination of Equations (4) and (8) gives:

(10) $$d\Delta G_{I}^{0}\left(\Phi ,T_{CE}\right)=0\;\mathrm{or}\;\Delta G_{I}^{0}\left(\Phi ,T_{CE}\right)=const.$$

This means that the grain boundary concentration is the same for all grain boundaries at *T_CE_*. 

**Figure 4:**
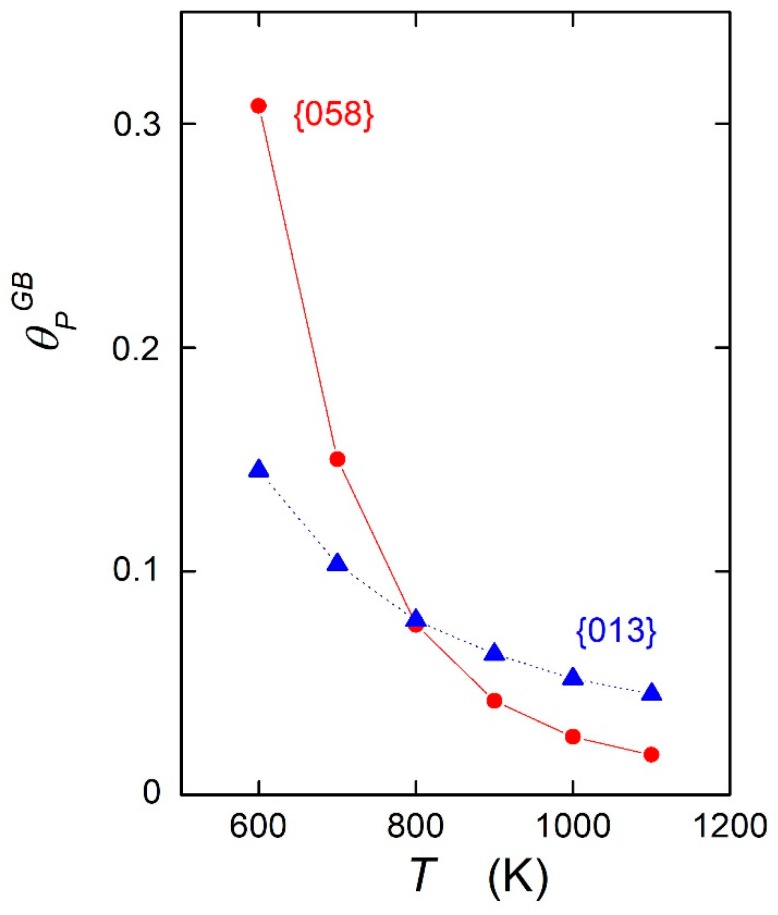
Temperature dependence of the phosphorus concentration at the {013} grain boundary (triangles) and at the {058} grain boundary (circles) calculated according to Equation (1). Data are from Reference [14].

As a consequence, the sign of the differences in chemical composition which occur at temperatures lower than $T_{CE}$ is reversed at temperatures above *T_CE_*. This can be documented by the reversed character of phosphorus segregation at {013} and {058} grain boundaries, as shown in Figure 1. A direct comparison is represented in Figure 4. An experimental indication of this effect was reported already many years ago for silicon segregation measured by AES at the individual grain boundaries of stainless steel showing a maximum Si concentration at the {013}, {012}, and {023} special grain boundaries, although the opposite behavior was expected [17]. Let us mention that such a crossing of the dependences cannot be observed when the entropic term is neglected. Accepting that the {013} grain boundary is special and the {058} grain boundary is general [1], Figure 4 clearly suggests that it is incorrect to characterize the grain boundaries by the level of the solute segregation. Such a characterization must be made exclusively on the basis of the values of the standard enthalpy of grain boundary segregation [16].

## 4. Discussion and Consequences for Practical Applications

In the above analysis, we saw that the consideration or negligence of entropy in the quantification of grain boundary segregation as a representative for intergranular properties gives very different results. Large differences are apparent between the grain boundary concentrations determined in these two ways, and the enthalpy-entropy compensation effect cannot be considered if entropy is neglected. These differences led us to conclude that calculations of the grain boundary composition that neglect the entropy term are incorrect.

An incorrect determination of the grain boundary segregation can have important consequences for practical applications. It is known, for example, that phosphorus segregation at grain boundaries induces the temper embrittlement of ferritic steels, which can result in brittle fracture [18,19]. If the grain boundary concentration of phosphorus in a steel is determined incorrectly and without consideration of the entropy term, its value will be lower by tens of percentage points than the real value determined with the entropy term included. This may then result in an incorrect assessment of the fracture resistivity of the grain boundaries in steels as, for example, the ductile-brittle transition temperature is proportional to the grain boundary concentration of phosphorus [19]. An incorrect assessment of its value can then have fatal practical consequences if such material is applied and could be similar to the turbine disaster described in detail in Reference [20]. Let us also note for completeness that the grain boundary segregation in binary systems is discussed here, while steels are complex multicomponent alloys. However, in many practical tasks, ferritic steels can be considered as pseudobinary systems [21]. Similarly, steels can be embrittled by impurities such as sulfur, tin, antimony, tellurium, selenium, and hydrogen [19]. 

Grain boundary segregation of impurities in steels also affects other materials properties controlled by interfaces [2]. Phosphorus accumulated at grain boundaries may accelerate void formation during creep [22]. Similarly, the grain boundary segregation of this impurity increases the propensity of steels to undergo intergranular stress corrosion cracking in solutions containing nitrate ions [23].

As mentioned in the introduction, grain boundary segregation can also have an important effect on the reduction of grain boundary mobility [19] and, consequently, on the recrystallization temperature and stabilization of nanocrystalline structures [3]. However, even here we meet problems with neglecting the entropy term [3] which can, for example, evoke doubts about the quantitative correctness of the model calculations for generating thermodynamic stability maps [24].

To assess the segregation effect on material behavior correctly, we must know the precise value of the grain boundary concentration of an impurity. An incorrect determination of the grain boundary concentration can thus result in misleading practical conclusions. For example, the systematically lower values of grain boundary concentrations of phosphorus as determined in Section 3.1. without considering the entropy term (Figure 1) may incorrectly predict the effect of grain boundary segregation on the above-mentioned processes. If such values and resulting consequences are considered as being valid, an incorrect estimate of the materials properties and of the lifespan of technological parts could lead to fatal problems. 

## 5. Conclusions

Both model calculations, as well as phenomena such as the temperature and concentration dependences of grain boundary segregation, clearly prove that the entropy term is irreplaceable in all considerations of grain boundary segregation. This conclusion is supported by several examples: (1) the comparison of the temperature dependence of phosphorus and silicon segregation at two differently oriented grain boundaries, calculated with and without the entropic term; and (2) the enthalpy-entropy compensation effect and its consequence in changing the character of grain boundary segregation as compared with the two defined grain boundaries. These examples clearly illustrate that the entropy of grain boundary segregation cannot be neglected in any treatment that deals with this phenomenon. As the entropy of segregation can be obtained from experimental studies on the temperature dependence of grain boundary chemistry at present, it is a great challenge to find new approaches for theoretical calculations of this parameter in order to make significant progress in understanding the phenomenon of grain boundary segregation. 

## Figures and Tables

**Table 1:** Parameters of phosphorus and silicon segregation at {013} and {058} grain boundaries [14].

GB	$\Delta H_{P}^{0}\;(\mathrm{kJ}\;{{\mathrm{mol}}^{-}}^{1})$	$\Delta S_{P}^{0}\;(J\;{{\mathrm{mol}}^{-}}^{1}\;{K^{-}}^{1})$	$\Delta H_{Si}^{0}$ (kJ mol^−^^1^)	$\Delta S_{Si}^{0}$ (J mol^−^^1^ K^−^^1^)
{013}	−14.7	41.4	−5	−2
{058}	−38	12	−11	−7
